# Gene expression profiling in Pekin duck embryonic breast muscle

**DOI:** 10.1371/journal.pone.0174612

**Published:** 2017-05-04

**Authors:** Tie-Shan Xu, Li-Hong Gu, Wei Huang, Wan-Liang Xia, Yun-Sheng Zhang, Ya-Ge Zhang, Guang Rong, Kyle Schachtschneider, Shui-Sheng Hou

**Affiliations:** 1 Tropical Crop Genetic Resource Research Institute, Chinese Academy of Tropical Agricultural Sciences, Danzhou, Hainan, P.R. China; 2 Institute of Animal Science & Veterinary, Hainan Academy of Agricultural Science, Haikou, P.R. China; 3 Institute of Animal Science, Chinese Academy of Agricultural Sciences, Beijing, P.R. China; 4 Department of Radiology, University of Illinois at Chicago, Chicago, Illinois, United States of America; Northwest A&F University, CHINA

## Abstract

Lean-type Pekin duck is a breed gained through long-term selection and great effort has been exerted to understand the mechanisms underlying increased muscle yields. However, the genes involved in Pekin duck embryonic breast muscle development have not been explored to date. In this study, we investigated gene expression profiles in Pekin Duck embryonic breast muscle at hatched day 13 (E13), E19, and E27 using RNA-seq. In total, we produced 519,312,178 raw reads resulting in 497,348,158 high-quality reads after filtering. The mapping, distribution of reads along annotated genes, and consistency across replicates demonstrates the high quality of the RNA-seq data used in this study, allowing us to continue with the downstream analysis. Significantly fewer differentially expressed genes (DEGs) were identified between E13 and E19 (203 DEGs) compared to E27 and E19 (2,797 DEGs). Many DEGs highly expressed in E19 are involved in metabolic processes and cell division. KEGG analysis showed many pathways associated with fat development were significantly enriched for DEGs highly expressed in E27. These results provide a basis for the further investigation of the mechanisms involved in Pekin duck embryonic breast muscle development.

## 1. Introduction

Skeletal myogenesis is a multistep process in which mesodermal precursor cells are selected to form myoblasts that are withdrawn from the normal cell cycle and subsequently differentiate into myotubes [1, 2]. These myotubes further differentiate into muscle fibers [3, 4] during the final stage of embryonic development. The formation, determination, and terminal differentiation of muscle cells are mainly governed by a network of four muscle regulatory factors (MRFs): myogenic factor 5 (*MYF5*), muscle-specific regulatory factor 4 (*MRF4*), myoblast determination protein (*MYOD*), and *myogenin* [5]. What’s more, many other genes such as sine oculis homeobox homologue (*SIX1-SIX6*), eyes absent (*EYA*), and muscle enhance factor 2C (*MEF2C*) act as skeletal myogenesis regulators [6–8]. Obviously, skeletal myogenesis is tightly regulated by a complicated transcriptional network.

Pekin duck is world-famous for its fast growth, but its skeletal muscle yield is lower than that of other high lean yield ducks [9]. To increase the skeletal muscle yield of Pekin ducks, the Chinese Academy of Agricultural Sciences started a breeding program in the 1990s to produce a new strain of lean-type Pekin duck characterized by increased skeletal muscle yield. Subsequently, our lab performed many studies to explore the mechanisms underlying the increased muscle yield in lean-type Pekin ducks. First, we identified genes involved in breast muscle development in postnatal Pekin duck using suppression subtractive hybridization (SSH) and RNA-seq technologies [9, 10]. Subsequently, we performed an association analysis, identifying associations between *MUSTN1*, *MSTN*, *IGF1*, and *FOXO3* genes with breast muscle development in postnatal Pekin duck [11–13]. In addition, we determined the developmental characteristics of breast muscle in Pekin duck embryos. These results show that the embryonic breast muscle development is fastest at 19 days after hatching (E19) and that E19 is the crucial transition point for Pekin duck breast muscle [14]. Based on the above findings, we profiled microRNAs (miRNAs) in Pekin duck embryonic breast muscle, providing insights into the miRNA landscape of embryonic duck breast muscle and a foundation for further investigation of the roles of miRNAs in duck skeletal muscle development [15]. However, the role and expression profile of genes in Pekin duck embryonic breast muscle is still largely unknown.

Here, we constructed three mRNA libraries from Pekin duck embryonic breast muscle at E13, E19, and E27. Gene expression profiles were determined via RNA-seq data and subsequent bioinformatics analysis in this study. We further investigated the roles of differentially expressed genes (DEGs) for the development and regulation of Pekin duck embryonic breast muscle. The results presented here, in combined with our previous results, provide further insights into the molecular mechanism underlying breast muscle development in Pekin duck.

## 2. Materials and methods

### 2.1. Tissue collection, RNA isolation, and cDNA library construction

Three full-sib Pekin duck embryos per time point at stage E13, E19 and E27 were collected in sterile physiological saline immediately after removal from the hatching eggs. Breast muscle samples were frozen in liquid nitrogen and stored at −80°C. Total RNA was isolated from all samples using the RNAiso plus kit (Takara, Dalian, China) following the manufacturer's instructions. The RNA quality was analyzed by 1.0% agarose gel electrophoresis and spectrophotometric absorption at 260 nm in a Nanodrop ND-1000^®^ Spectrophotometer. The total RNA was used for construction of cDNA libraries and subsequent sequencing according to Xu et al. [10]. Additional RNA from each sample was stored at −80°C for validation of consistency across replicates using qRT-PCR technology.

### 2.2. Quality evaluation and filtering of raw data

To ensure the RNA-seq data was of high enough quality for downstream analysis, we carried out adapter trimming and filtering of the raw reads to decrease the data noise. Specifically, we filtered the raw reads to remove: i) adapter contamination, ii) reads in which unknown bases were more than 5% of the read, and iii) low quality reads (q-value < 20).

### 2.3. Mapping of high-quality reads to the duck reference genome

We mapped the high-quality reads to the duck reference genome (http://www.ensembl.org/Anas_platyrhynchos/Info/Index) using SOAPaligner/SOAP2 software [16] with no more than 2 mismatches. Basic mapping statistics and read distributions across the duck genome and annotated genes were determined to evaluate the randomness of the distribution.

### 2.4. Consistency of RNA-seq data

To determine how consistent the gene expression results were across individuals from the same time point, we calculated the correlation coefficient of gene expression among replicates based on the RPKM values.

### 2.5. Calculation of gene expression levels

The expression level of each gene covered by our RNA-seq data was calculated using the reads per kilobase per million reads (RPKM) method [17]. The detected genes were then classified into bins according to their RPKM values.

### 2.6. Hierarchical clustering analysis (HCA) of significantly expressed genes

We first defined the significantly expressed genes as the genes expressed in all of the three time points (E13, E19 and E27) with their p-values and correct p-values (FDR) less than 0.05. Then, we performed clustering analysis of the genes to identify their expression tendencies by using hierarchical clustering schemes [18]. The detailed procedure of hierarchical clustering schemes is as follow. First, we dealt the expression tendency of each gene as a cluster. Then, we calculated the similarity of each cluster and combined the most similar two groups as a new cluster. The two steps above were repeated until all the expression tendencies of significantly expressed genes were grouped into one group.

### 2.7. Analysis of DEGs

Genes differentially expressed between two time points were identified using the method described by Audic and Claverie [19]. Genes were considered differentially expressed if: I) the false discovery rate (FDR) [20] was less than 0.001, II) the P-value was less than 0.01, and III) expression between two time points differed by more than 1 fold change.

### 2.8. Real time quantitative polymerase chain reaction (qRT-PCR)

qRT-PCR was performed using the SYBR PrimeScript RT-PCR Kit (TaKaRa, Dalian, China) with SYBR Green dye to validate the RNA-seq results as previously described [10]. Primers used in this study are presented in Table 1.

**Table 1:** The primers used in this paper.

Primer^1^	Sequence (5' to 3')	Tm (°C)
*AQP4*	TCGGTCTTCTACATTCTCG	50.4
	ACCAGTGACATCGTTTCG	51.0
*ACTA1*	TCCTCACGCTCAAGTATCCC	57.4
	AGGTCACGGCCAGCCAAGTC	59.6
*GAS2*	TCCTTCGCCCTCACCAAC	55.9
	TCCAACACGGACCATCAC	55.6
*TFCP2L1*	GGAGAAGAAGACCACCCA	51.9
	TAAGTCAGCACCCGAAAA	51.9
*ENPP6*	CACGGCTACGACAACGAG	55.2
	AGACATCCACGGACCTGA	52.8
β-actin	GCTATGTCGCCCTGGATTTC	55.50
	CACAGGACTCCATACCCAAGAA	57.30

### 2.9. Data deposition

Data described in this study is available in the NIH Short Read Archive (SRA) under accession number SUB1749540.

### 2.10. Funding

Financial support of this work was provided by “the earmarked fund for China Agriculture Research System (CARS-43-1, CARS-43-42)”

### 2.11. Ethics statement

All sample collection and subsequent experiments were approved by the Ethical and Animal Welfare Committee of Beijing, China. Birds were slaughtered using the electric shock method followed by the oral bloodletting method within 30 seconds to ameliorate their suffering.

## 3. Results and discussion

### 3.1. Sequencing and filtering of raw data

RNA-seq data was produced using RNA obtained from Pekin duck embryonic breast muscle samples at E13, E19 and E27. In total, 519,312,178 raw reads were obtained (accumulated length of 51,931,217,800 bp), including 171,658,529 for E13, 179,524,180 for E19, and 168,132,911 for E27. After filtering, a total of 497,348,158 high-quality reads (accumulated length of 49,734,815,800 bp) remained and were used for downstream analysis, including 164,148,468 for E13, 171,960,228 for E19, and 161,239,462 for E27. Overall, ~95.77% of raw reads were defined as high-quality reads and the average GC content of high-quality reads was 50.44% (Table 2). Much higher percentage of high-quality reads were obtained in this study compared to most of the other similar RNA-seq studies in human [21–23], mouse [24], sheep [25], freshwater fish [26] and our previous study [10]. The results above indicate the RNA-seq data used in this study are sufficient in sequencing depth and read quality.

**Table 2:** The basic information for the RNA-seq data used in this study.

Samples		Raw reads	Clean readas	Clean bases	Q20(%)	GC content
E13-1	R1	27,194,108	26,117,221	2,611,722,100	96.04	49.14
	R2	27,564,349	26,117,221	2,611,722,100	94.75	49.16
E13-2	R1	29,447,497	28,443,337	2,844,333,700	96.59	49.84
	R2	29,736,892	28,443,337	2,844,333,700	95.65	49.75
E13-3	R1	28,624,299	27,513,676	2,751,367,600	96.12	52.02
	R2	29,084,224	27,513,676	2,751,367,600	94.60	52.01
Sum		171,658,529	164,148,468	16,414,846,800	95.63	50.32
E19-1	R1	32,303,029	31,275,793	3,127,579,300	96.82	50.22
	R2	32,876,898	31,275,793	3,127,579,300	95.13	50.34
E19-2	R1	27,047,785	26,128,160	2,612,816,000	96.60	50.03
	R2	27,316,424	26,128,160	2,612,816,000	95.65	50.17
E19-3	R1	29,810,308	28,576,161	2,857,616,100	95.86	48.72
	R2	30,188,211	28,576,161	2,857,616,100	94.66	48.87
Sum		179,524,180	171,960,228	17,196,022,800	95.79	49.73
E27-1	R1	29,617,001	28,574,483	2,857,448,300	96.48	51.21
	R2	29,967,995	28,574,483	2,857,448,300	95.35	51.20
E27-2	R1	26,099,881	25,170,725	2,517,072,500	96.44	51.20
	R2	26,387,174	25,170,725	2,517,072,500	95.39	51.30
E27-3	R1	27,806,025	26,874,523	2,687,452,300	96.65	51.34
	R2	28,262,197	26,874,523	2,687,452,300	95.09	51.37
Sum		168,132,911	161,239,462	16,123,946,200	95.90	51.27
Total		519,312,178	497,348,158	49,734,815,800	95.77	50.44

### 3.2. Mapping the high-quality reads to the duck reference genome

The high-quality reads were then mapped to the duck reference genome. The basic statistical results are summarized in Table 3. The mapping ratio for each sample ranged from 81.79% to 86.51%, with an average mapping ratio of 84.25%. For each sample, around 90% of annotated duck genes (~14,000 out of 15,697 genes) were covered by at least 1 read, and around 80% of annotated duck genes were covered by at least 5 reads.

**Table 3:** Mapping results of high-quality reads.

Samples	Mapping ratio %	Gene counts and percentage covered by reads (Out of 15,697 duck annotated genes)				
		Reads>0	Reads>1	Reads>2	Reads>5	Reads>10
E13-1	83.54	14202(90.48%)	13819(88.04%)	13398(85.35%)	12766(81.33%)	12142(77.35%)
E13-2	85.21	14659(93.39%)	14361(91.49%)	14058(89.56%)	13539(86.25%)	12992(82.77%)
E13-3	82.56	13842(88.18%)	13420(85.49%)	12960(82.56%)	12249(78.03%)	11570(73.71%)
E19-1	85.43	13991(89.13%)	13591(86.58%)	13132(83.66%)	12466(79.42%)	11797(75.15%)
E19-2	86.51	14646(93.30%)	14346(91.39%)	14025(89.35%)	13466(85.79%)	12978(82.68%)
E19-3	81.79	14562(92.77%)	14242(90.73%)	13901(88.56%)	13323(84.88%)	12777(81.40%)
E27-1	84.12	14216(90.57%)	13813(88.00%)	13449(85.68%)	12863(81.95%)	12252(78.05%)
E27-2	83.87	14067(89.62%)	13696(87.25%)	13277(84.58%)	12695(80.88%)	12065(76.86%)
E27-3	85.26	14200(90.46%)	13838(88.16%)	13414(85.46%)	12798(81.53%)	12246(78.01%)

**Fig 1:**
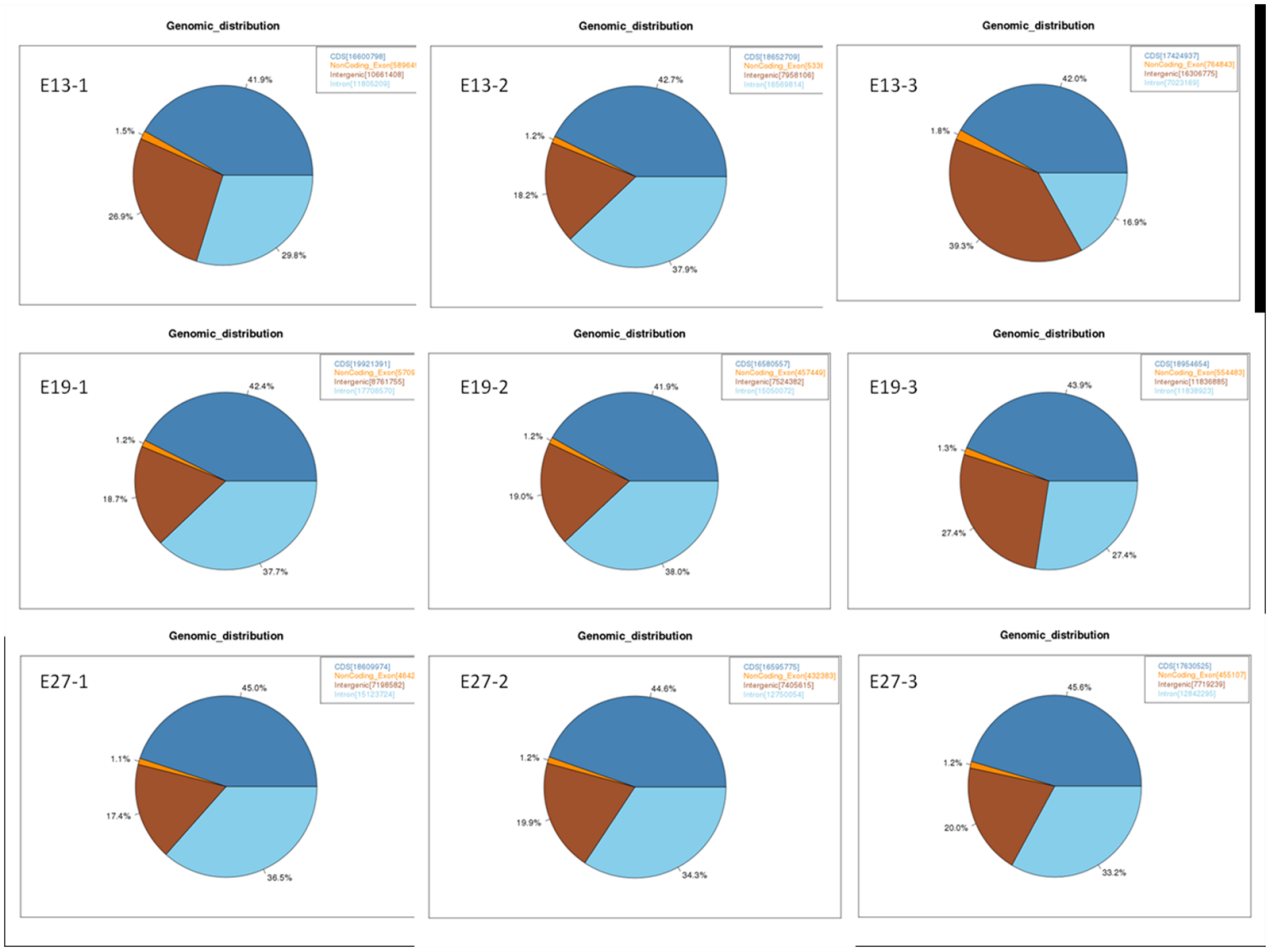
Distribution of mapped reads along the duck genome. E13-1, E13-2, and E13-3 denote the first, second, and third sample collected at E13, respectively; E19-1, E19-2, and E19-3 denote the first, second, and third sample collected at E19, respectively; E27-1, E27-2, and E27-3 denote the first, second, and third sample collected at E27, respectively.

To demonstrate the positional relationship between the mapped reads and annotated genes, we classified the mapped reads into several types such as the reads located in coding sequence (CDS), introns, intergenic regions, and noncoding exons (Fig 1). The majority of reads (43.33%, ranging from 41.9% to 45.6%) were located in annotated CDS, with 32.46% of reads (ranging from 16.9% to 37.9%) located in intronic regions. These results indicate the high quality of the RNA-seq data used in this study since most of the mapped reads were located in CDS regions. However, the proportion of mapped reads located in annotated CDS was relatively low. This may be due to the relatively low quality of the duck draft genome assembly. Indeed, many genes annotated in other model organisms are not annotated in the current duck genome, resulting in regions covering potentially unannotated genes being classified as intergenic regions. This is supported by the high proportion of aligned reads located in intergenic regions (average 22.98%). Therefore, it is expected that the proportion of reads mapped to intergenic regions will decrease and the proportion mapped to CDS will increase with continued improvement of the duck genome assembly.

**Fig 2:**
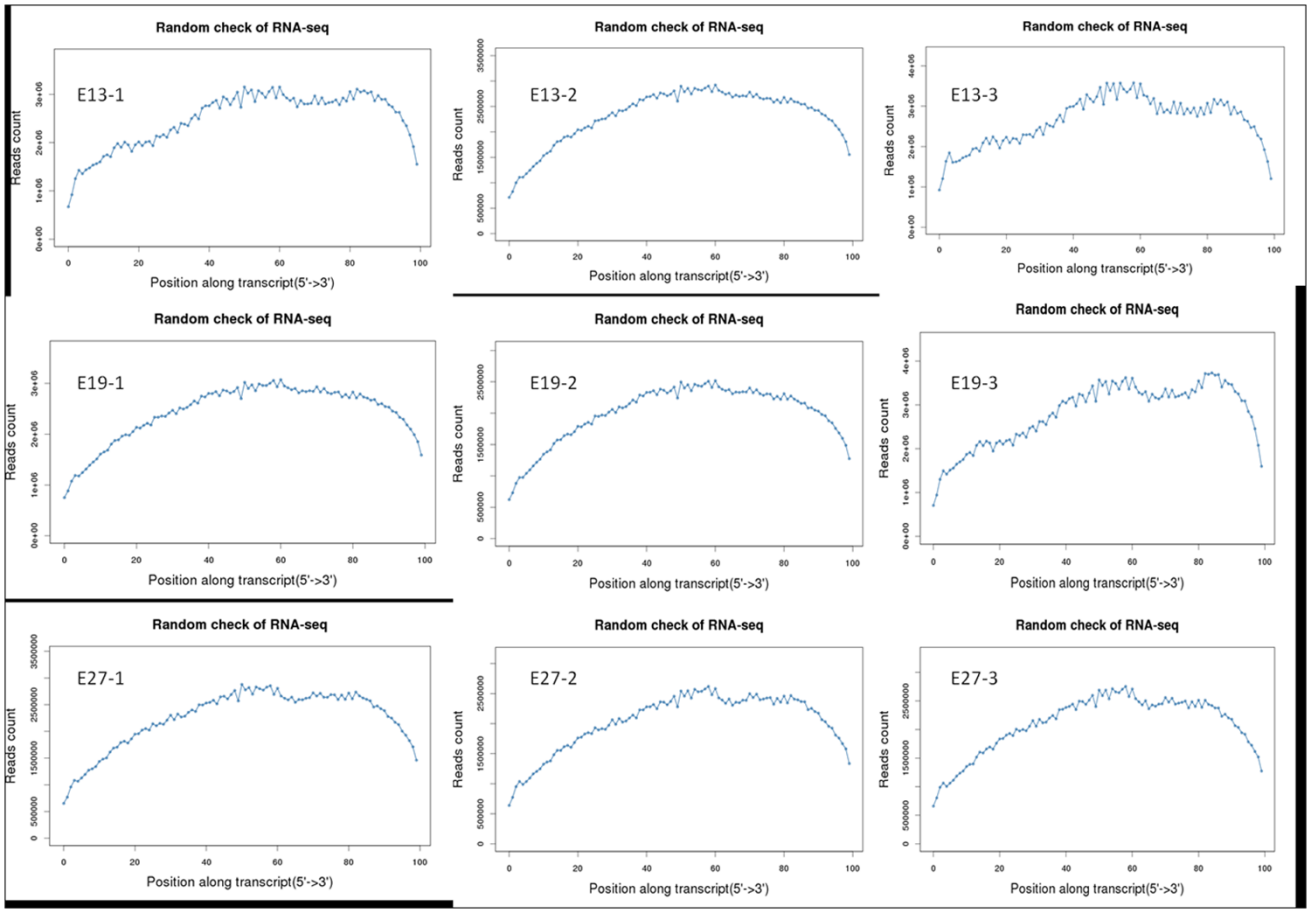
Distribution of mapped reads across annotated duck genes.

Larger RNA molecules must be fragmented into smaller pieces (200–500 bp) to be compatible with RNA-seq deep-sequencing technology during the RNA-seq library construction. Compared to other methods, RNA fragmentation provides more even coverage along the gene body, while reducing coverage at the 5′ and 3′ ends [27]. Therefore, we investigated the distribution of reads along annotated duck genes (Fig 2). Consistent with previous results, more reads mapped to the gene body than the 5′ and 3′ ends across all samples. This indicates that the read distribution across annotated genes is as expected, and that the RNA-seq data is suitable for downstream analysis.

### 3.3. Consistency across replicates

**Fig 3:**
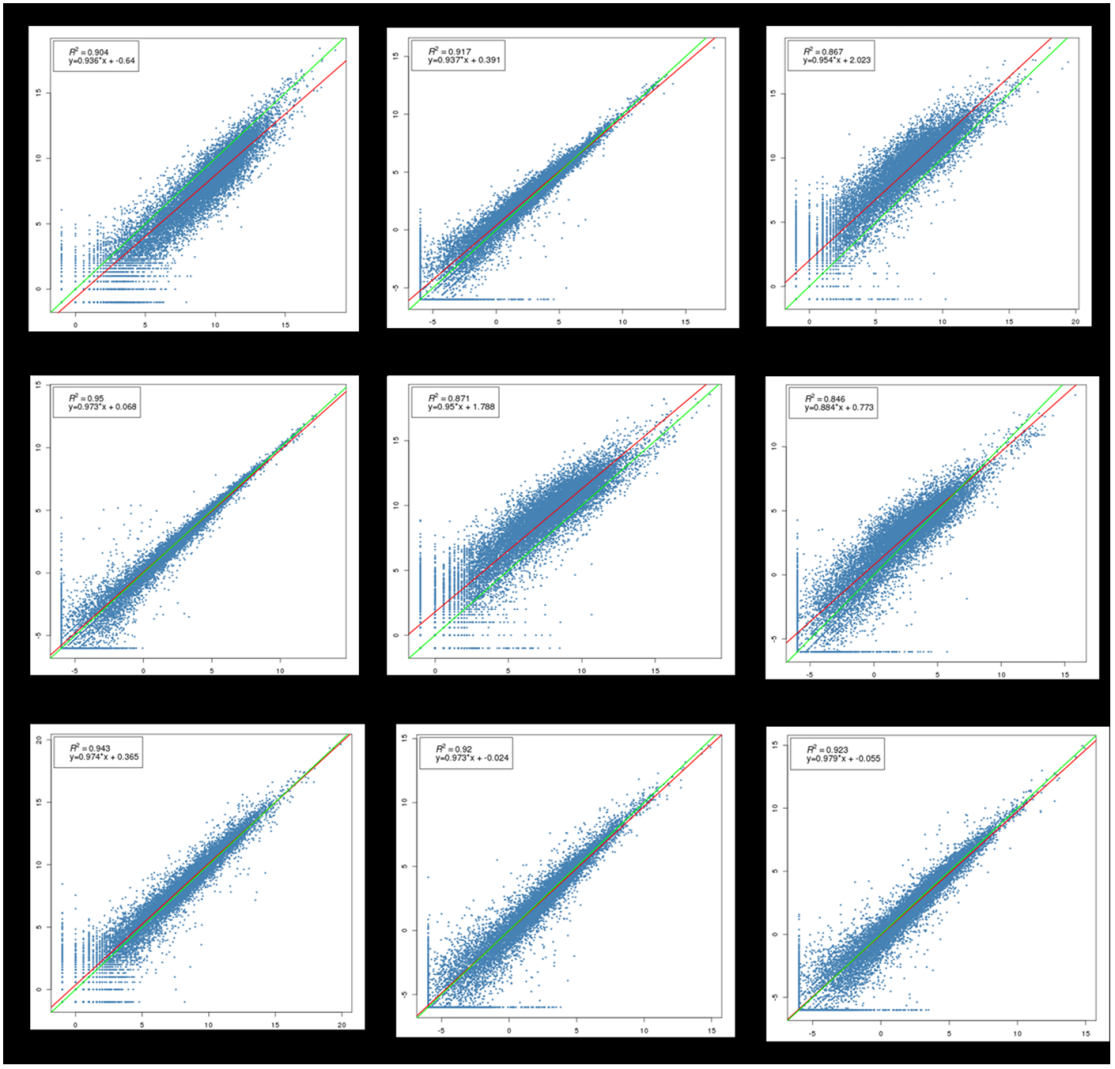
Consistency across replicates. E13-2_VS_E13-1, E13-3_VS_E13-1, and E13-3_VS_E13-2 describe comparisons between E13-2 and E13-1, between E13-3 and E13-1, and between E13-3 and E13-2, respectively; E19-2_VS_E19-1, E19-3_VS_E19-1, and E19-3_VS_E19-2 describe comparisons between E19-2 and E19-1, between E19-3 and E19-1, and between E19-3 and E19-2, respectively; E27-2_VS_E27-1, E27-3_VS_E27-1, and E27-3_VS_E27-2 describe comparisons between E27-2 and E27-1, between E27-3 and E27-1, and between E27-3 and E27-2, respectively; R^2^ denotes the correlation coefficient between the two samples; The equation denotes the linear regression equation of the two samples.

The consistency of the gene expression results across replicates at each time point is an important index for the reliability of RNA-seq data [27]. In this study, replicates were highly correlated, with an average correlation coefficient of 0.90 (ranged from 0.846 to 0.950) (Fig 3).

**Fig 4:**
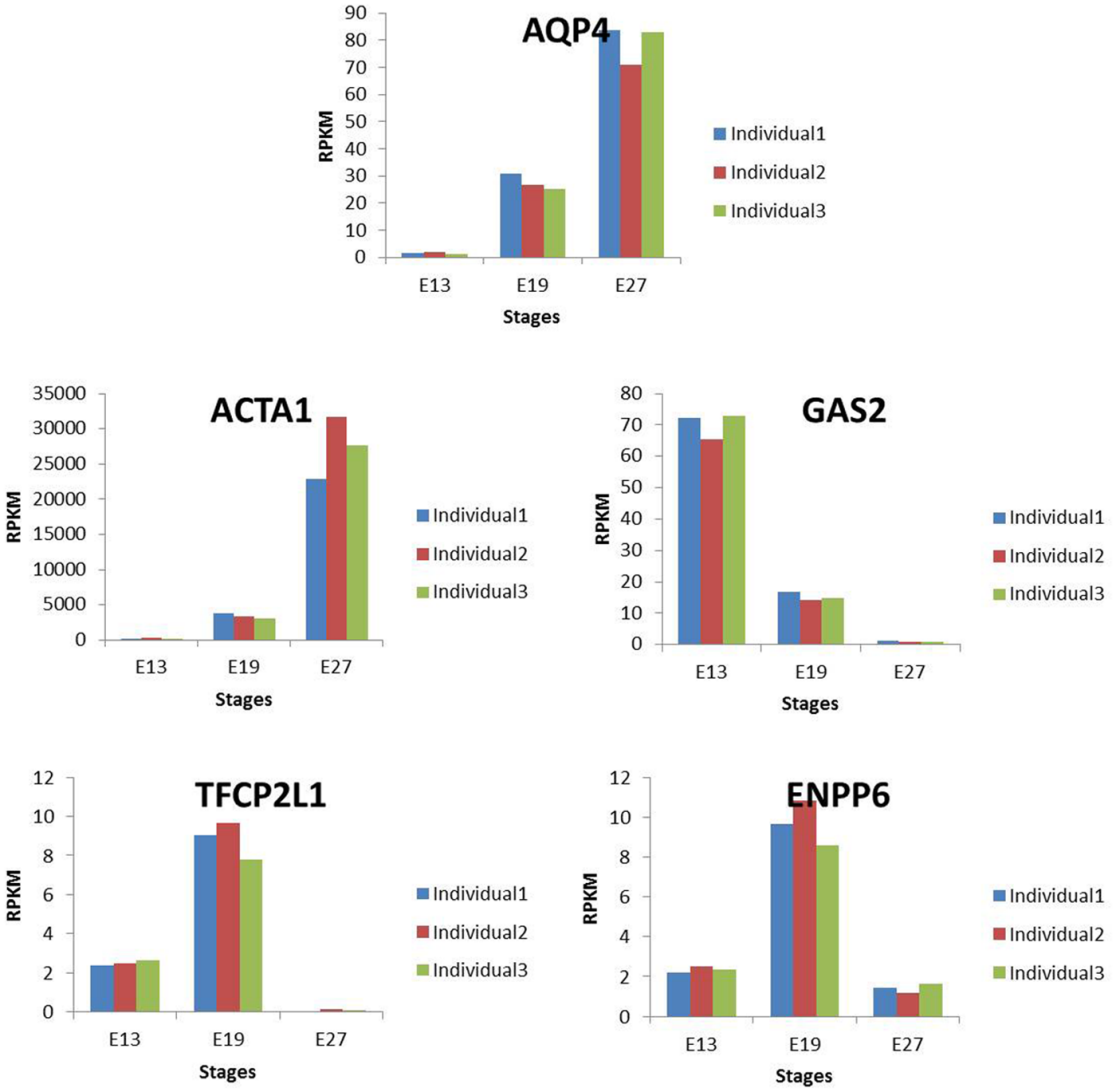
RNA-seq based expression of 5 genes across biological replicates at E13, E19 and E27.

**Fig 5:**
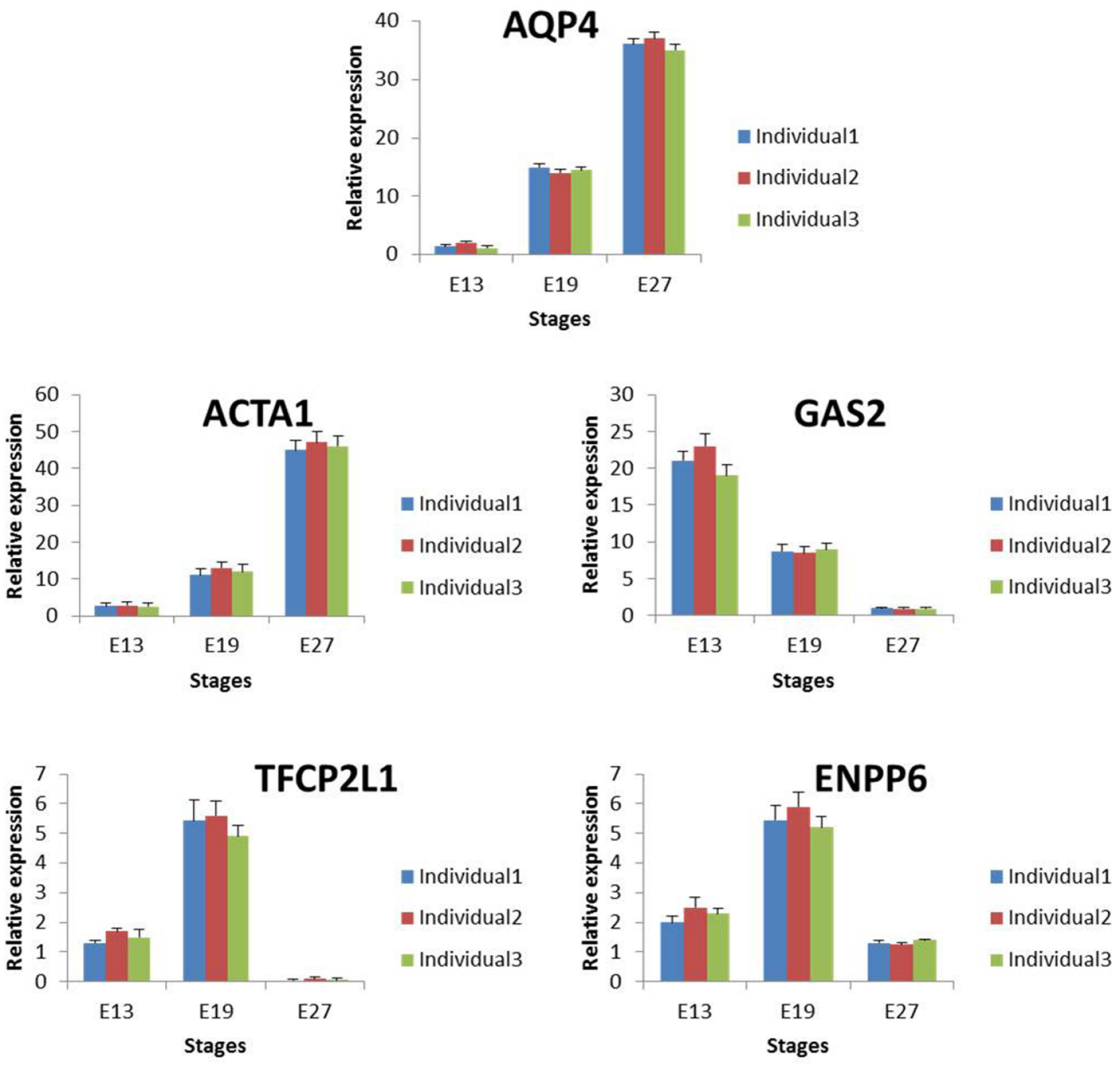
qRT-PCR based expression of 5 genes across biological replicates at E13, E19 and E27.

For an excellent RNA-seq experiment, highly consistent expression levels are expected across replicates. Based on this, we compared the expression levels of five genes (*APQ4*, *ACTA1*, *GAS2*, *TFCP2L1*, and *ENPP6*) across replicates using qRT-PCR and RPKM approaches. The results showed no significant differences in gene expression based on the RPKM values (Fig 4) or qRT-PCR results (Fig 5) across replicates. These results demonstrate the high quality of the RNA-seq data, in addition to demonstrating that the biological replicates used in this study are similar in genetic background.

### 3.4. Analysis of gene expression levels

**Fig 6:**
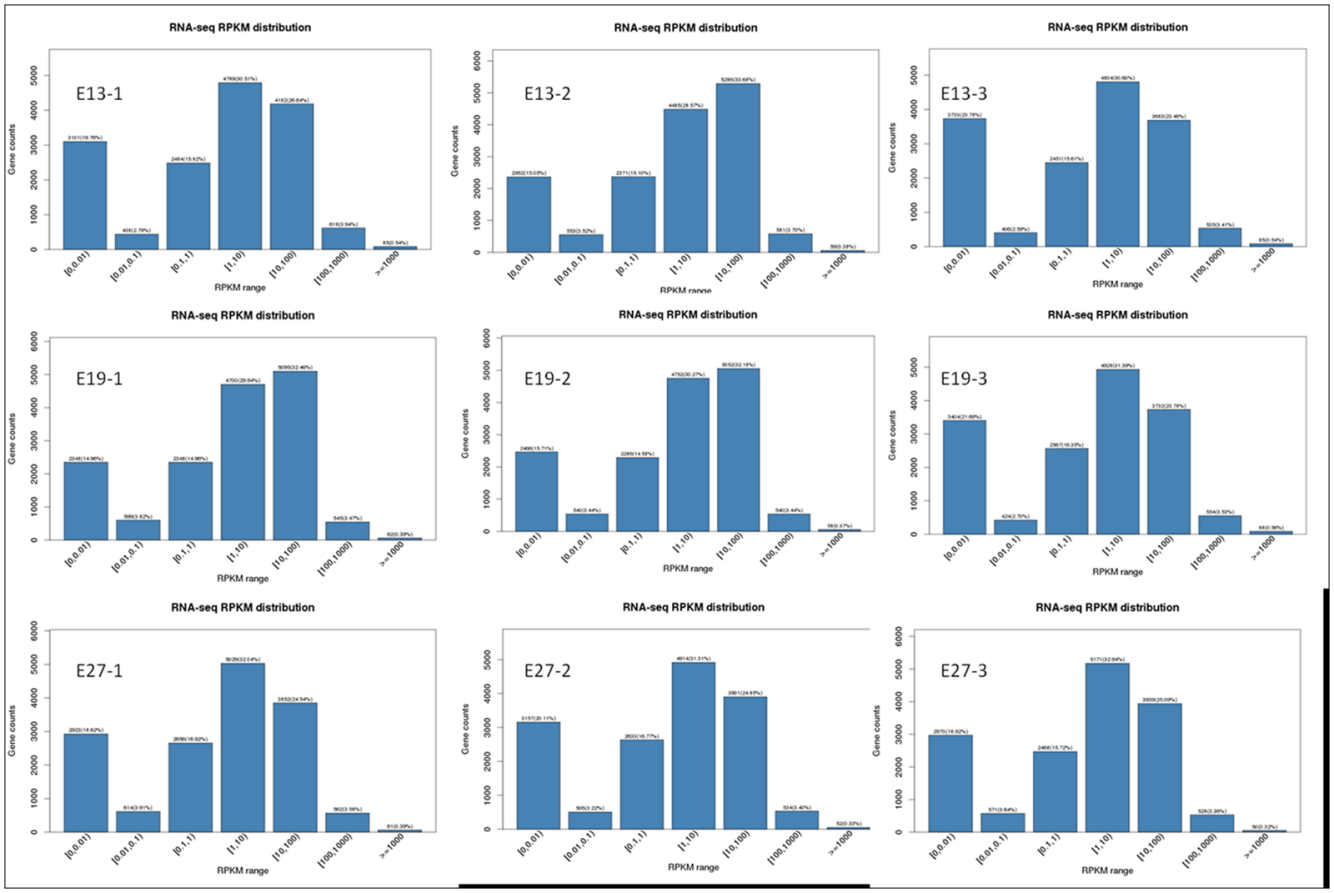
Analysis of gene expression levels.

We calculated gene expression levels for all samples using the RPKM method [17]. Expressed genes were classified into several bins according to the range of their RPKM values (Fig 6). In general, the percentage of genes with moderate RPKM values was higher than those with lower or higher RPKM values. For example, genes with RPKM values ranging from 1 to 10 and from 10 to 100 accounted for 30.51% (ranged from 28.57 to 32.94) and 26.64% (ranged from 23.46 to 33.68) of expressed genes respectively, which is greater than the percentage of the genes with RPKM values ranging from 0.01 to 0.1 and more than 1000 (average of 2.79% and 0.54%, respectively). This result demonstrates that most of the genes expressed in Pekin duck embryonic breast muscle are moderately expressed. However, lowly expressed genes (RPKM from 0 to 0.01) accounted for a relatively high percentage of the total genes (averagely 19.76%), suggesting many annotated genes are not expressed in Pekin duck embryonic breast muscle.

### 3.5. HCA of significantly expressed genes

**Fig 7:**
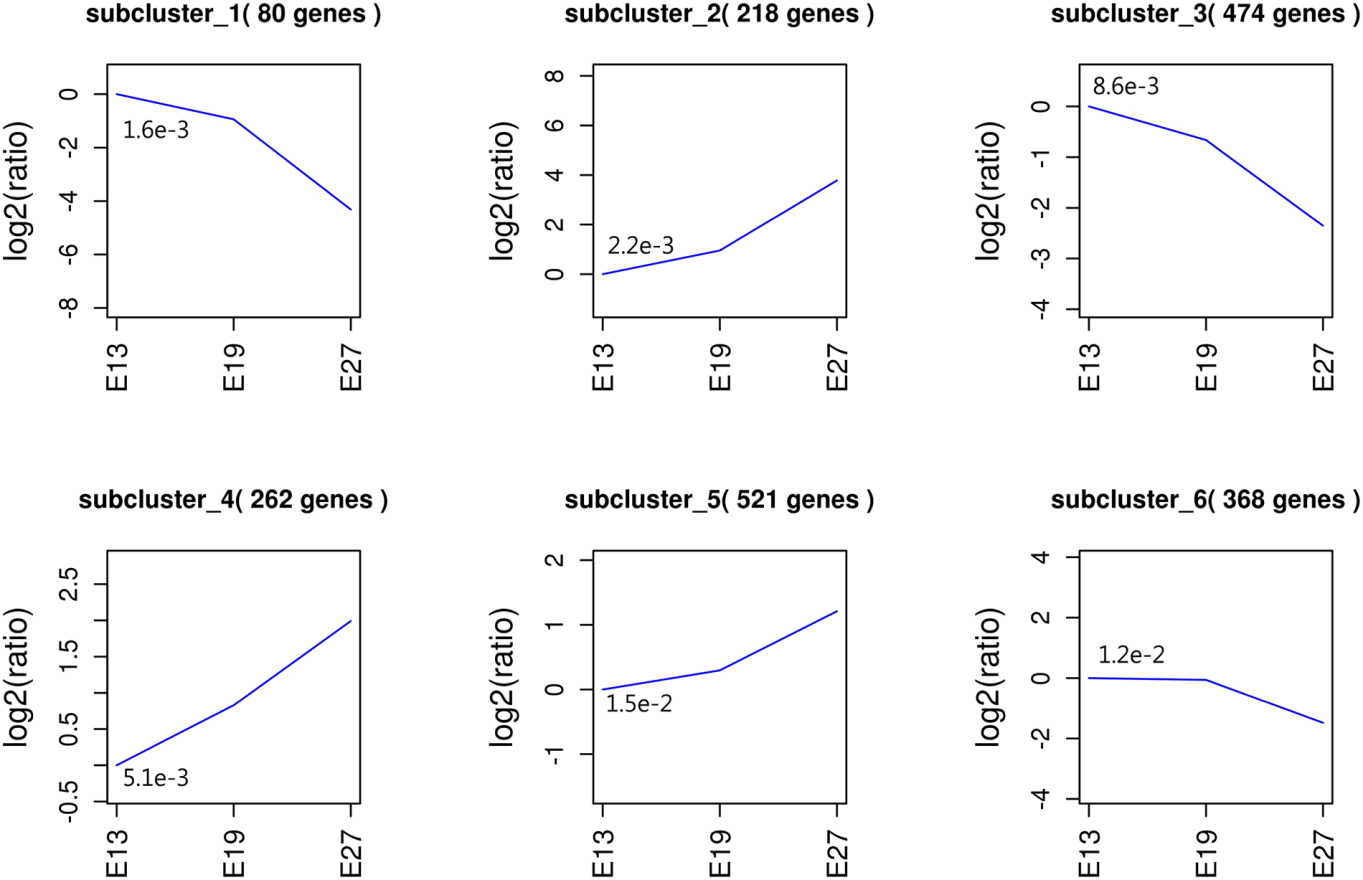
The expression profiles of 1,924 significantly expressed genes in E13, E19 and E27.

Total 1,924 genes were declared to be significantly expressed in E13, E19 and E27. To further narrow the target genes which harbor great significance among the declared 1,924 genes, we chose to use h-cluster methods to summarize the expression pattern of the genes and obtained six subclusters in the obtained final cluster (Fig 7). Genes in subcluster1, subcluster 3 and subcluster 6 showed similar expression tendencies with their expression continuous increase along with duck embryonic growth. In contrast, the expression tendencies in subcluster2, subcluster 4 and subcluster 5 decreased Continuously from E13 to E27.

### 3.6. Analysis of DEGs

**Fig 8:**
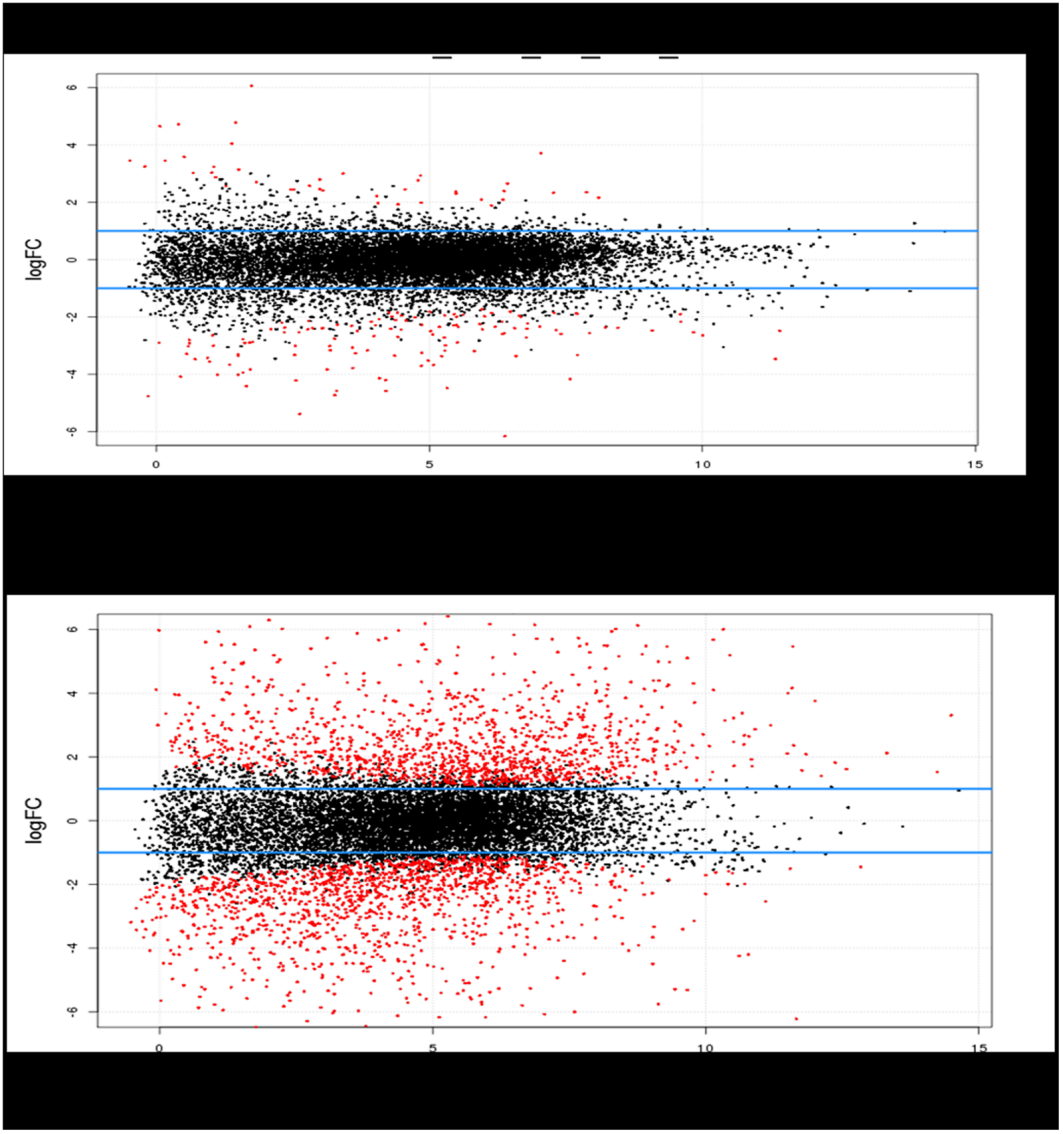
DEGs between E13 and E19, and between E27 and E19. Red dots represent DEGs between E13 and E19, or between E27 and E19; Blue horizontal lines indicate a 2 fold change in expression.

The mainobjective of this study was to identify DEGs involved in Pekin duck embryonic breast muscle development. The general landscape of DEGs is demonstrated in Fig 8. DEGs between E13 and E19 are provided in S1 Table, while DEGs between E27 and E19 are provided in S2 Table. Significantly less DEGs were identified when comparing E13 to E19 than when comparing E27 to E19. 203 DEGs were identified when comparing E13 to E19 (S1 Table), 149 of which displayed increased expression in the E19 samples, compared to 54 displaying increased expression in the E13 samples. Log2 Fold Changes (log2 E13/E19) ranged from -8.72 (*SNAP91*) to 6.07 (*GAP43*). When comparing the E27 and E19 samples, 2,797 DEGs were detected, 1,437 of which displayed increased expression in the E19 samples, compared to 1,360 displaying increased expression in the E27 samples (S2 Table). Log2 Fold Changes (log2 E27/E19) ranged from -8.03 (*LOC101803953*) to 9.13 (*KLHL38*). As the fastest growth rate for Pekin duck embryonic breast muscle occurs at E19 [14], DEGs with increased expression at E19 likely play crucial roles in promoting breast muscle development in Pekin duck, while DEGs with reduced expression at E19 may negatively regulate breast muscle development in Pekin duck.

### 3.7. GO analysis of DEGs

**Fig 9:**
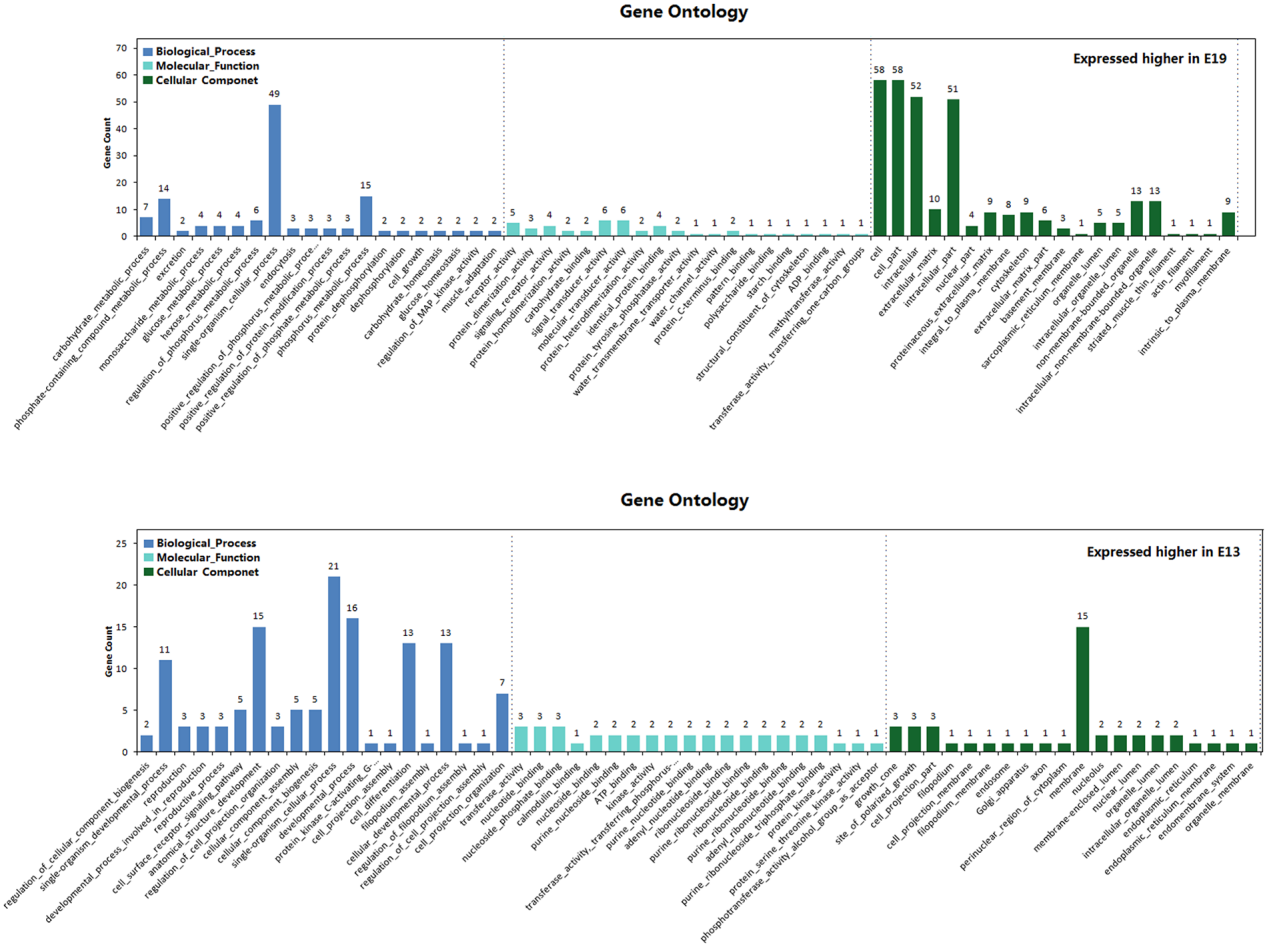
The 20 most significantly enriched GO terms of DEGs between E13 and E19.

**Fig 10:**
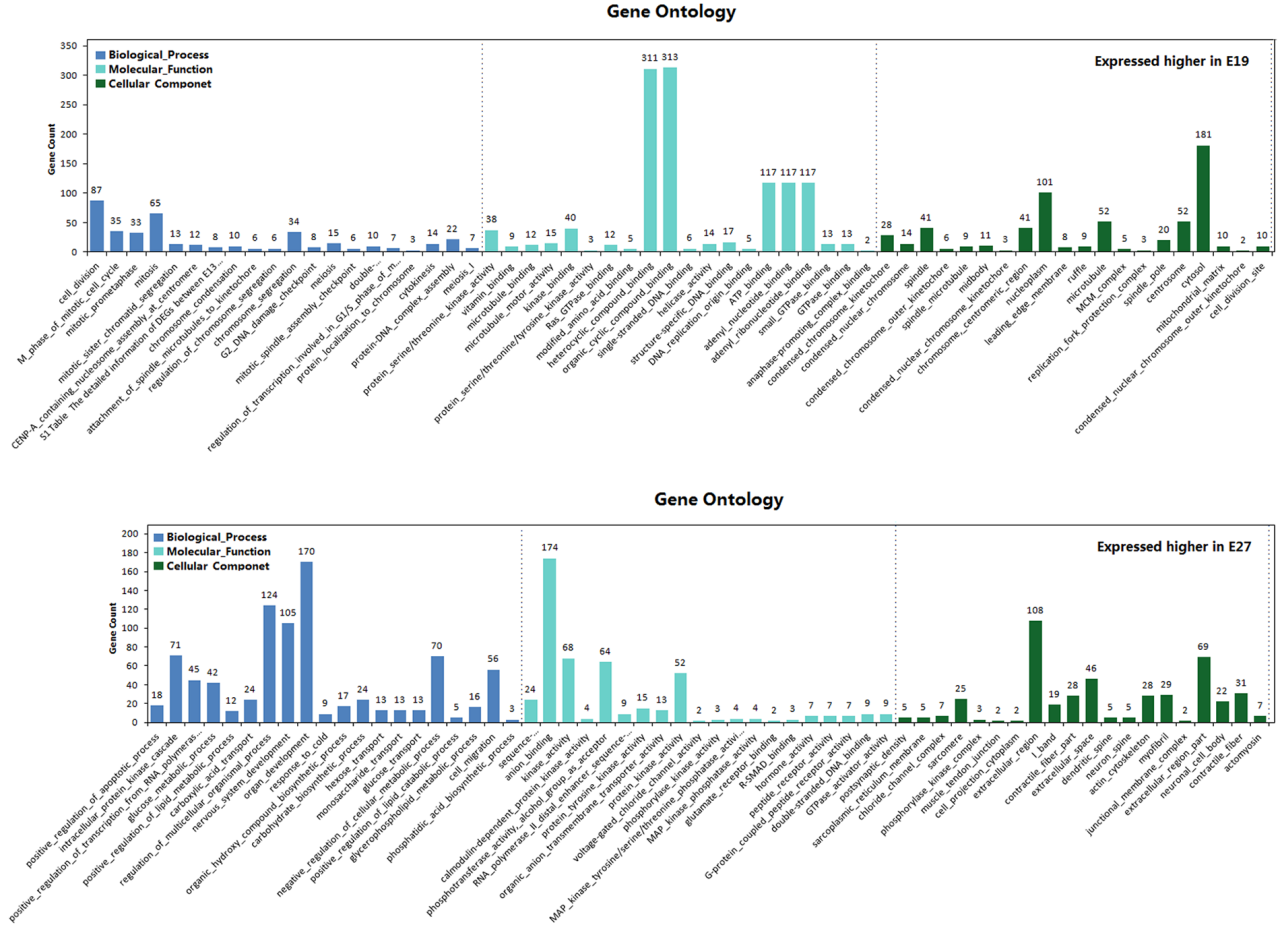
The 20 most significantly enriched GO terms of DEGs between E27 and E19.

Gene Ontology (GO), the *de facto* standard in gene functionality description, is used widely for functional annotation and enrichment analysis [28]. Here, we performed separate GO enrichment analysis for DEGs highly and lowly expressed at E19 compared to the other two time points. We then selected the 20 most significantly enriched GO terms to compare the functional differences of DEGs across time points. The results are presented in Figs 9 and 10, S3 and S4 Tables and are divided into four groups. I) DEGs highly expressed at E19 compared to E13. Biological processes enriched in this category suggest embryonic breast muscle cells may be more metabolically active at E19 than E13. Enriched processes include carbohydrate metabolic process, phosphate containing compound metabolic process, regulation of phosphorus metabolic process, monosaccharide metabolic process, glucose metabolic process, hexose metabolic process, positive regulation of phosphorus metabolic process, positive regulation of protein modification process, and phosphorus metabolic process. Cellular component enrichment analysis suggests products of genes in this category were predominately intracellular. This is based on enrichment of a number of cellular components, including cell, cell part, intracellular, nuclear part, intracellular part, integral to plasma membrane, and cytoskeleton. II) DEGs highly expressed at E13 compared to E19. The top 5 enriched GO terms for this category were associated with developmental process and cell differentiation. III) DEGs highly expressed at E19 compared to E27. The majority of enriched GO terms were involved in cell division, while the enriched molecular function categories indicate genes in this category are involved in material binding, including heterocyclic compound binding (311 genes enriched), organic cyclic compound binding (313 genes enriched), ATP binding (117 genes enriched), adenyl nucleotide binding (117 genes enriched), and adenyl ribonucleotide binding (117 genes enriched). These results suggest increased expression of genes involved in developmental processes and material binding are required at E19 due to the increased Pekin duck embryonic breast muscle growth rate compared to E27 [14]. IV) DEGs highly expressed at E27 compared to E19. Many enriched Go terms in this category are involved in adaptation to new environments, including nervous system development, regulation of multicellular organismal process, organ development, and response to cold. These results suggest that the genes highly expressed at E19, specifically genes associated with cell differentiation, cell division, and material binding, represent candidates for investigating the mechanism underlying increased breast muscle growth rates at E19.

### 3.8. KEGG analysis of DEGs

KEGG (Kyoto Encyclopedia of Genes and Genomes) is a knowledge base for systematic analysis of gene functions, linking genomic information with higher order functional information [29]. We performed KEGG pathway enrichment analysis separately for the same four groups of DEGs used for GO term analysis (S5 and S6 Tables). Two pathways were significantly enriched for DEGs highly expressed at E13 compared to E19, as well as DEGs highly expressed at E19 compared to E13. However, 16 and 14 pathways were significantly enriched for DEGs highly expressed at E27 compared to E19 and for DEGs highly expressed at E19 compared to E27, respectively.

Of the pathways enriched for DEGs highly expressed at E27 compared to E19, the pathway containing the most DEGs was the metabolic pathway (167 genes), indicating a high level of metabolic activity in Pekin duck breast muscle at E27. In addition, many pathways were associated with fat development, including PPAR signaling pathway, adipocytokine signaling pathway, glycerophospholipid metabolism, glycerolipid metabolism, and fatty acid degradation. Previous reports have identified decreased myofibres and satellite cell mitotic activity in the breast muscle of late-term turkey embryos [30], indicating skeletal muscle may undergo atrophy during the final days of incubation. A developmental transition from atrophy in late-term embryo breast muscle to hypertrophy in neonates has also been observed in ducks [31, 14]. In addition, other studies have shown adipocytes share a common mesenchymal cell origin with skeletal muscle cells [32, 33] and that mesenchymal cells lacking exposure to certain signaling factors differentiate into fat cells [34]. Thus, these results suggest fat metabolism becomes more active in breast muscle at E27 than at E19 due to skeletal muscle atrophy during the final days of incubation.

A number of pathways enriched for DEGs highly expressed at in E19 compared to E27 were involved in cell development, such as DNA replication, cell cycle, biosynthesis of amino acids, gap junction, non-homologous end-joining, and base excision repair, consistent with previous reports indicating Pekin duck breast muscle undergoes its quickest growth phase at E19 [14].

The KEGG enrichment analysis allows us to identify DEGs highly expressed at E27 that play a role in fat development, providing candidate genes for further investigation of the mechanisms underlying skeletal muscle and fat development at E27.

### 3.9. Important DEGs involved in duck muscle development and fat deposition

In the current study, we identified many DEGs involved in duck muscle development and fat deposition, including NR1H3, ADIPOR2, SOCS3, ACADM, FABP5, RXRA, FABP3, STK11, CD36, CYP8B1, ACSL4, ACSL1, UBB, HADHB, FABP4, PRKAA2, HADHA, ACSBG2, ACOX1, ACADL, ECI2, mTOR, CYP27A1, NFKB1, PRKAB2, and SLC27A1.

**Fig 11:**
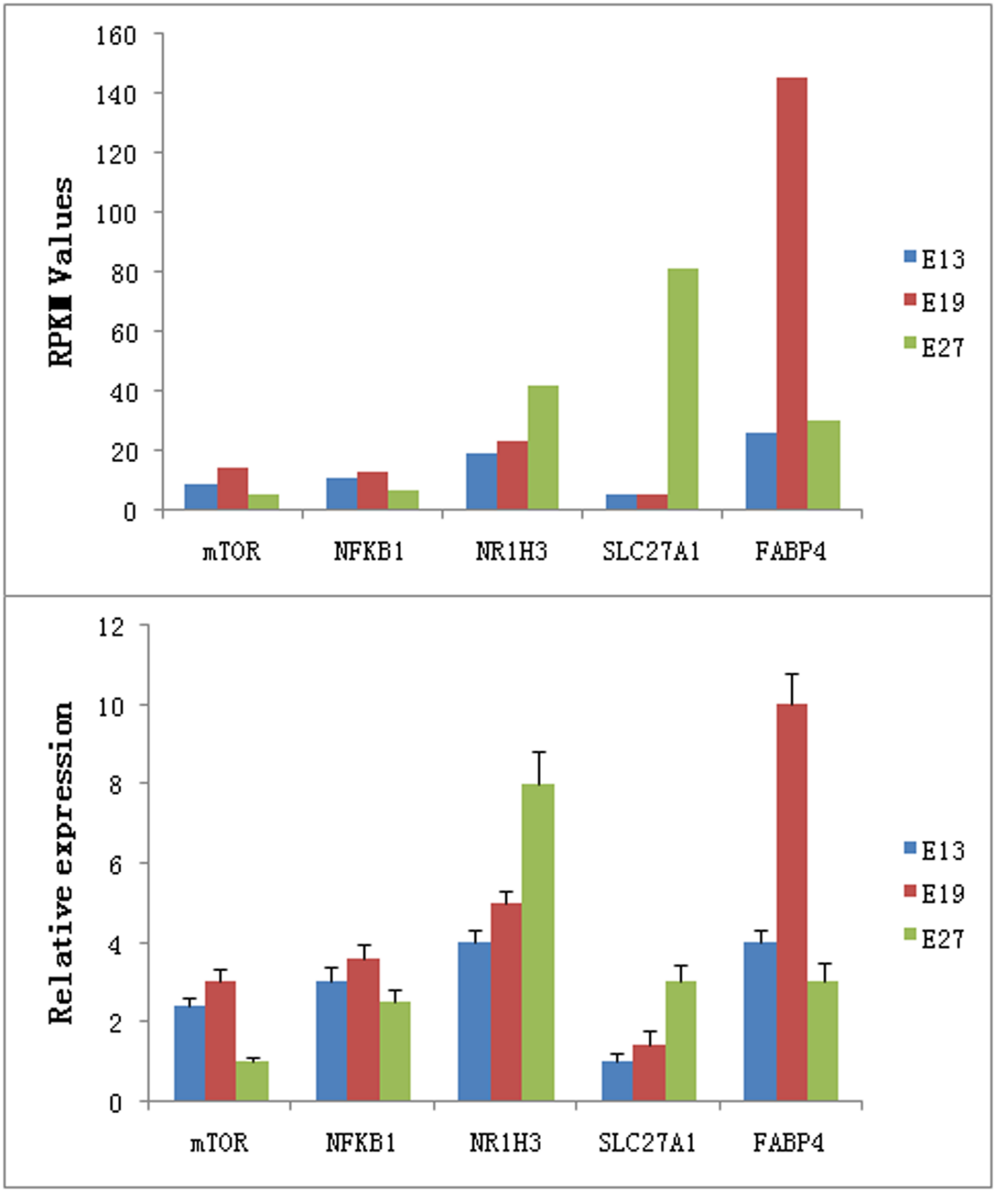
The relative expression values and RPKM values of five genes involved in muscle development and fat deposition.

To validate the expression of the above-mentioned DEGs, we compared the expression levels of the five most important DEGs involved in muscle development and fat deposition (mTOR, NFKB1, NR1H3, FABP4, and SLC27A1) using qRT-PCR (Fig 11). mTOR (mammalian target of rapamycin) is a crucial promoter of skeletal muscle hypertrophy and can prevent muscle atrophy *in vivo* through the Akt/mTOR pathway [35]. NFKB1 (nuclear factor kappa-B, subunit 1) is another crucial promoter of skeletal mass development whose disruption inhibits skeletal muscle atrophy [36]. In this study, both mTOR and NFKB1 were expressed at higher levels at E19 than E13 or E27. Since Pekin duck embryonic breast muscle grows fastest at E19 [14], we concluded that mTOR and NFKB1 play similar roles in Pekin duck skeletal muscle development as they do in other species. Previous studies have demonstrated that NR1H3 and SLC27A1 are positively associated with fat deposition [37, 38]. In this report, expression of both NR1H3 (Nuclear receptor subfamily 1, group H, member 3) and SLC27A1 (solute carrier family 27A, member 1) increased continuously from E13 to E27, suggesting that fat deposition increases as the embryo grows. FABP4 (fatty acid binding protein 4) was also shown to attenuate PPARγ and adipogenesis by Garin-Shkolnik et al. [39]. Our results also demonstrate FABP4 is likely a negative regulator of Adipogenesis due to the fact that its expression peaks at E19. Thus, we not only identified a number of DEGs involved in muscle development and fat deposition, but also confirmed the expression levels of five genes critical for muscle development and fat deposition.

## 4. Conclusions

In conclusion, we investigated expression profiles of genes in duck embryonic breast muscle at E13, E19, and E27 using RNA-seq. The RNA-seq data used in this study were found to be sufficient in sequencing depth and read quality for downstream analysis following evaluation of the read quality and confirmation of consistency across replicates. Differential expression analysis detected hundreds to thousands of DEGs between the E13 and E19, and E27 and E19 time points. A number of cell division related GO terms (M phase of mitotic cell cycle, cell division, mitosis, mitotic prometaphase, etc.) and KEGG pathways (DNA replication, Cell cycle, Gap junction, etc.) were significantly enriched for DEGs highly expressed at E19 compared to the other two time points. In addition, many pathways (PPAR signaling pathway, Adipocytokine signaling pathway, and Fatty acid degradation) enriched for DEGs highly expressed at E27 compared to E19 are involved in fat development. Finally, many DEGs involved in skeletal muscle development and fat deposition, including mTOR, NFKB1, NR1H3, FABP4, and SLC27A1, were identified in this study.

## Supporting information

S1 Table Detailed information of DEGs between E13 and E19. (XLSX)

S2 Table Detailed information of DEGs between E27 and E19. (XLSX)

S3 Table GO terms enriched for DEGs between E13 and E19. (XLSX)

S4 Table GO terms enriched for DEGs between E27 and E19. (XLSX)

S5 Table KEGG pathways enriched for DEGs between E13 and E19. (XLSX)

S6 Table KEGG pathways enriched for DEGs between E27 and E19. (XLSX)
